# Digital versus Manual Tracing in Cephalometric Analysis: A Systematic Review and Meta-Analysis

**DOI:** 10.3390/jpm14060566

**Published:** 2024-05-25

**Authors:** Sameer Narkhede, Paritosh Rao, Veera Sawant, Sanpreet Singh Sachdev, Suraj Arora, Ajinkya M. Pawar, Rodolfo Reda, Luca Testarelli

**Affiliations:** 1 Department of Orthodontics and Dentofacial Orthopedics, School of Dentistry, D.Y. Patil Deemed to Be University, Navi Mumbai 400706, Maharashtra, India; sameer.narkhede@dypatil.edu (S.N.); paritoshrao724@gmail.com (P.R.); veera.sawant@dypatil.edu (V.S.); 2 Department of Oral Pathology and Microbiology, Bharati Vidyapeeth (Deemed to Be University) Dental College and Hospital, Navi Mumbai 400614, Maharashtra, India; sunpreetss@yahoo.in; 3 Department of Restorative Dental Sciences, College of Dentistry, King Khalid University, Abha 61421, Saudi Arabia; surajarorasgrd@yahoo.co.in; 4 Department of Conservative Dentistry and Endodontics, Nair Hospital Dental College, Mumbai 400034, Maharashtra, India; 5 Department of Oral and Maxillo-Facial Science, Sapienza University of Rome, Via Caserta 06, 00161 Rome, Italy; rodolfo.reda@uniroma1.it

**Keywords:** orthodontics, cephalometry, skeletal malocclusion, artificial intelligence

## Abstract

**Background:** Over the years, various researchers have attempted to compare digital cephalometry with the conventional manual approach. There is a need to comprehensively analyze the findings from the earlier studies and determine the potential advantages and limitations of each method. The present systematic review aimed to compare the accuracy of digital and manual tracing in cephalometric analysis for the identification of skeletal and dental landmarks. **Methods:** A systematic search was performed using the keywords “Digital” AND “Manual” AND “Cephalometry” to identify relevant studies published in the English language in the past decade. The electronic data resources consulted for the elaborate search included the Cochrane Central Register of Controlled Trials (CENTRAL), MEDLINE, CINAHL, EMBASE, PsycINFO, Scopus, ERIC, and ScienceDirect with controlled vocabulary and free text terms. **Results:** A total of *n* = 20 studies were identified that fulfilled the inclusion and exclusion criteria within the timeframe of 2013 to 2023. The data extracted from the included articles and corresponding meta-analyses are presented in the text. **Conclusions:** The findings of the present systematic review and meta-analysis revealed trends suggesting that digital tracing may offer reliable measurements for specific cephalometric parameters efficiently and accurately. Orthodontists must consider the potential benefits of digital cephalometry, including time-saving and user-friendliness.

## 1. Introduction

Cephalometry is a valuable diagnostic tool used in dentistry to assess craniofacial structures and aid in the diagnosis, treatment planning, and evaluation of orthodontic and orthognathic cases [1]. This technique involves the analysis of cephalometric radiographs, which provide detailed measurements and visual representations of the skull, jaws, and soft tissues. The use of cephalometry in dentistry dates back to the early 20th century, when researchers began to explore the relationship between facial structures and malocclusions [2]. Over the years, cephalometric analysis techniques have evolved with advancements in imaging technology and the development of standardized landmarks and measurements.

Cephalometry plays a crucial role in various aspects of dentistry, particularly in orthodontics. It provides orthodontists with valuable information for accurate diagnosis, treatment planning, and evaluation of treatment outcomes. By analyzing cephalometric radiographs, clinicians can assess the skeletal and dental relationships, identify growth patterns, and predict the potential for future growth.

There are two main types of cephalometric analysis: two-dimensional (2D) and three-dimensional (3D) cephalometry. Two-dimensional cephalometry involves the analysis of lateral cephalometric radiographs [3]. It provides measurements and visual representations of the craniofacial structures in two dimensions, allowing for the assessment of skeletal and dental relationships, as well as soft tissue profiles. Three-dimensional cephalometry utilizes advanced imaging techniques such as cone-beam computed tomography (CBCT) to create three-dimensional models of the craniofacial complex [4]. This type of analysis provides more detailed information about the spatial relationships of the structures, allowing for a more comprehensive assessment of the patient’s condition.

Cephalometric analysis relies on the identification of specific landmarks on the radiographs and the measurement of various parameters. These landmarks can be categorized into skeletal, dental, and soft tissue landmarks, each serving a specific purpose in the analysis [5,6]. The use of cephalometry for craniofacial assessment has been an integral part of orthodontic practice [7,8,9]. Conventionally, the technique involves manual tracing of the anatomical landmarks by superimposing transparent tracing papers on the lateral cephalograms to geometrically calculate certain craniofacial measurements [10]. The method has often been described as tedious, time-consuming, subjective, variable, and susceptible to errors [11,12,13].

The reliance on manual tools and materials in traditional cephalometry signifies methodological consistency across studies. However, it is worth noting that this approach is not without limitations. Manual tracing is inherently prone to inter-observer variability and subjectivity, as different individuals may interpret and trace cephalograms with varying degrees of precision [12,13]. The meticulous use of standardized tools helps mitigate some of these challenges, but it remains essential to acknowledge the potential for human error in manual cephalometric analyses.

Recent developments in digital technology in almost every field have introduced a new era in cephalometry [14]. The cephalometric analysis can now be performed using computerized software that automatically identifies and measures the anatomical landmarks, thereby efficiently providing more consistent assessments [6,15]. This minimizes the scope of human error, observer bias, and the time required for analysis while improving the validity and reproducibility of the results [15,16].

Digital cephalometry, often facilitated by specialized software and electronic devices, offers advantages such as increased efficiency, reproducibility, and the potential for three-dimensional analyses. The transition from manual to digital methods represents a paradigm shift in cephalometric analysis, introducing the capability for automated landmark identification and measurements, which may address some of the limitations associated with manual tracing.

The contemporary landscape of cephalometric analysis has been significantly influenced by the proliferation of digital technologies, with various software applications playing a pivotal role in facilitating precise and efficient assessments. The diverse range of software utilized across the studies in this systematic review reflects the evolving nature of digital cephalometry and the exploration of different platforms to enhance diagnostic capabilities.

Even so, it is equally important to critically evaluate the accuracy of the so-called “automatic” cephalometric assessment, as it relies on the automatic detection of landmarks by pre-trained software [16,17]. Over the years, various researchers have attempted to compare digital cephalometry with the conventional manual approach. There is a need to comprehensively analyze the findings from the earlier studies and determine the potential advantages and limitations of each method.

In this context, the present systematic review aims to compare the accuracy of digital and manual tracing in cephalometric analysis for the identification of skeletal and dental landmarks. The review has the objectives of analyzing the current state of knowledge in this domain and contributing to the ongoing evolution of digital cephalometry.

## 2. Materials and Methods

The present systematic review and meta-analysis were performed in accordance with Preferred Reporting Items for Systematic Review 2020 (PRISMA 2020) [18,19], and the protocol was registered in the PROSPERO database with reference ID: CRD42023452625 [20]. A systematic search was performed using the keywords “Digital” AND “Manual” AND “Cephalometry” to identify relevant studies published in the English language in the past decade (1 January 2013 to 31 July 2023). The electronic data resources consulted for the elaborate search included the Cochrane Central Register of Controlled Trials (CENTRAL), MEDLINE, CINAHL, EMBASE, PsycINFO, Scopus, ERIC, and ScienceDirect with controlled vocabulary and free text terms.

### 2.1. Eligibility Criteria

Studies using manual tracing and digital tracing techniques for cephalometric analysis, irrespective of the software, were considered eligible for inclusion in the present review. These included clinical trials, in vivo studies, randomized clinical trials, controlled clinical trials, non-randomized clinical trials, quasi-experimental studies, non-experimental studies, cohort studies, and cross-sectional studies. Only those studies with cephalometric radiographs of good quality without any artifacts and having fully intact permanent central incisors and first permanent molars and no craniofacial deformities were included.

**Figure 1:**
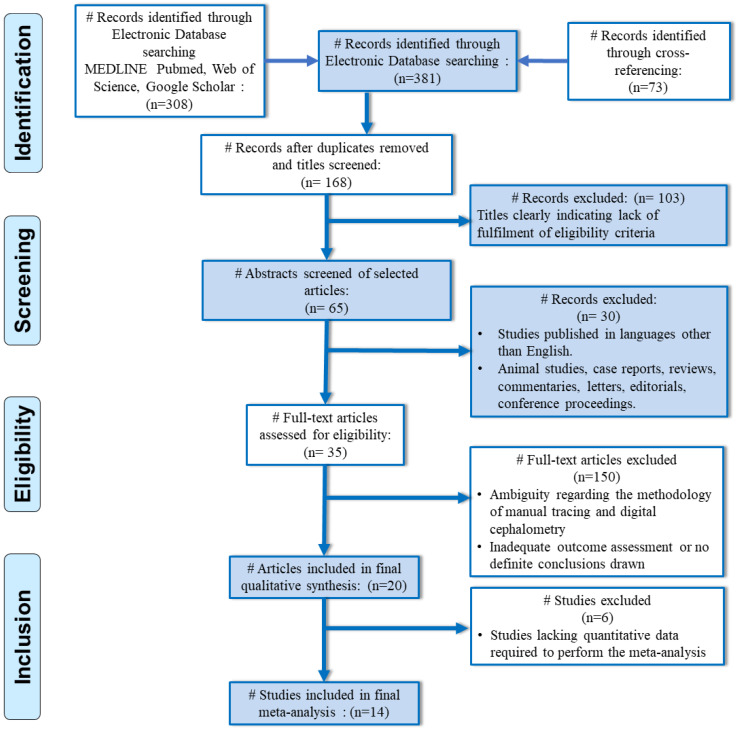
PRISMA flow diagram indicating the selection process of the articles in the present systematic review.

Studies involving cephalometric analysis of individuals with impacted teeth in the anterior region, prosthetic restoration of the central incisors, previous orthodontic treatment or orthognathic surgery, or cleft lip and palate syndromes were excluded from the review. Review reports, case series, in vitro, animal studies, and single intervention studies without the comparative group were excluded. Figure 1 denotes the selection process for the articles in the present systematic review.

### 2.2. Data Extraction

The author name, year, and country of the publication were recorded. The details pertaining to the study design, including the study settings, sample size, sampling technique, and demographic characteristics of the samples, were noted. Details related to digital cephalometry include the amount of exposure, the magnification of the radiographs, and the software used for cephalometric analysis.

The outcomes included either or all of the following outcomes using manual tracing techniques as compared to digital tracing techniques for cephalometric analysis:

1. Angular measurements—SNA, SNB, ANB, IMPA, Interincisal angle, SN-MP, SN-PP, MMA, and Gonial angle
2. Linear measurements were recorded—anterior cranial base (N-S), mandibular length (Go-Me), maxillary length (ANS to PNS), and LAFH—lower anterior facial height. (ANS to Me)

The conclusive findings reported by the authors were also recorded.

### 2.3. Data Reporting

The extracted qualitative data is planned to be reported primarily in the form of tables. The data concerning the temporal and geographical distribution of the studies has been depicted in the form of bar charts and map charts, respectively. The quantitative data and its subsequent meta-analysis have been narratively described, followed by a summarized depiction in the form of forest plots.

### 2.4. Assessments of the Risk of Bias and Quality

A simplified version of the NIH (National Institutes of Health) Quality Assessment Tool for Observational Cohort and Cross-Sectional Studies was adopted to evaluate the risk of bias and the methodological quality of the included papers, as they presented the results of cross-sectional studies [21]. The judgment of “Unsure” was reported for the specific item of the questionnaire for which information was not available in the manuscript. The quality of studies scoring more than five out of eight “Yes” was considered “Good”, the quality of studies that ranged from three to five “Yes” was considered “Fair”, and the quality of studies with less than three “Yes” was considered “Poor”.

### 2.5. Statistical Analysis for Quantitative Synthesis

Review Manager (RevMan) 5.3 was used for statistical analysis. Meta-analysis was conducted on the studies that provided information on similar outcomes, irrespective of the software used for digital tracing. The combined results were expressed as mean and standard deviation for the continuous data at 95% confidence intervals (CIs), and *p* < 0.05 was considered significant. Chi-square and Tau-square were used to assess whether the observed difference was homogeneous or heterogeneous among the studies. Statistical heterogeneity was assessed by the I2 test at α = 0.10. The I square statistic (I2) represents the percentage of the variability in effect estimates that is due to heterogeneity. I2 is the proportion of observed dispersion of results from different studies included in a meta-analysis that is real rather than spurious.

Heterogeneity was considered statistically significant if *p* < 0.05. For I2 > 50%, the random-effects model was applied. Subgroup analysis was performed to reduce the sources of clinical heterogeneity among the studies. Also, the statistical significance was set at a *p*-value (two-tailed) < 0.05. The detection of publication bias using funnel plots was carried out for studies exceeding 10 in number for each outcome assessed.

## 3. Results

**Figure 2:**
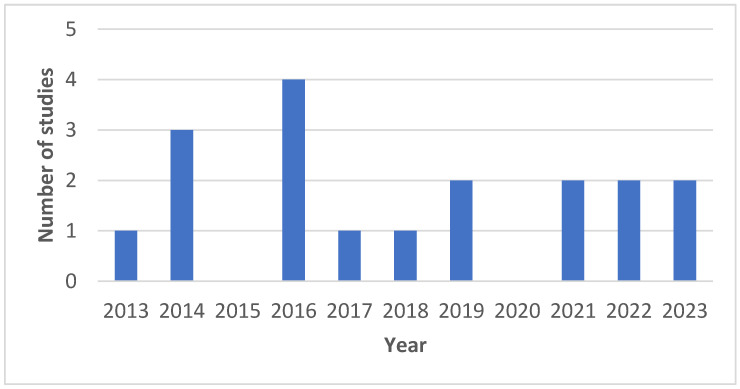
Year-wise distribution of the studies.

A total of *n* = 20 studies were identified fulfilling the inclusion and exclusion criteria within the timeframe of 2013 to 2023 [22,23,24,25,26,27,28,29,30,31,32,33,34,35,36,37,38,39,40,41]. The year-wise distribution of these studies is depicted in Figure 2. Findings from these studies are comprehensively summarized in Table 1. All the studies were cross-sectional comparative studies that used digital cephalometry as an intervention and manual tracing as a control. The quality assessment of all the articles is summarized in Table 2.

### 3.1. Narrative Synthesis

**Figure 3:**
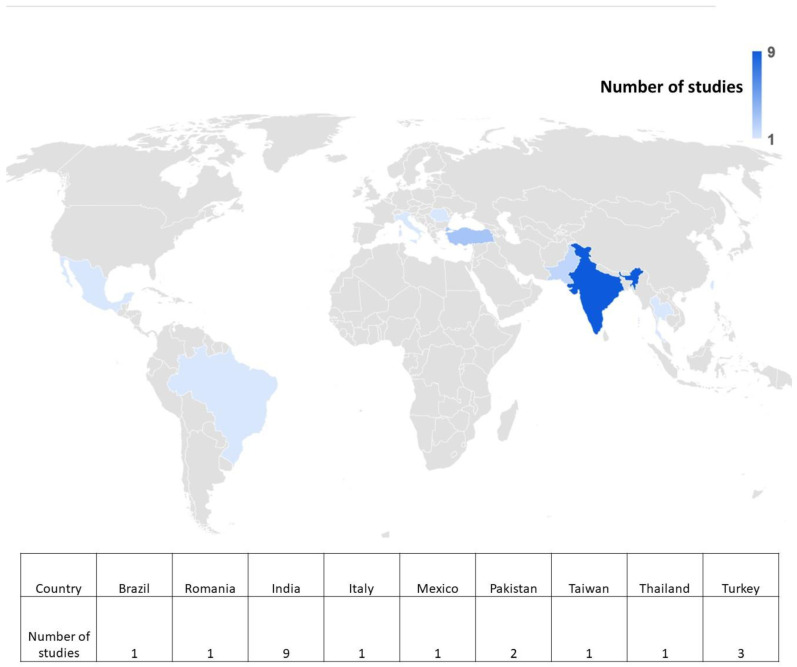
Geographic distribution of the studies conducted across the various countries.

All studies were conducted in different parts of the world, with nine in India, three in Turkey, two in Pakistan, and one study each in Taiwan, Brazil, Italy, Romania, Thailand, and Mexico, respectively (Figure 3). All the included studies showed a cross-sectional study design. Overall, 1559 subjects were included in this systematic review. Different software was used for digital tracing of lateral cephalograms, such as NemoCeph, SmileCeph, Orthalis, Dolphin Imaging, FACAD, FALA System, AutoCEPH, Carestream Dental, TrophyDicom, OnyxCeph, OneCeph, SATM CephNinja, Cephalopoint, and View Box.

The sample size across the majority of studies ranged from 20 to 150, with the exception of the study by Lindner et al. [28], which comprised 400 subjects in the Taiwanese population. All the Indian studies had a sample size of 20 to 50 subjects, except for Khattri et al. [40], who had a sample size of 100 subjects. Given the larger population size of India as well as the ethnic diversity across its various states, it is essential to conduct studies with relatively larger sample sizes across the different geographical areas to ensure that the results obtained can be extrapolated to the general population.

Since the age estimation methods are concerned with identifying the pubertal growth spurt, most of the studies included age groups of patients that spanned across the pre-pubertal, pubertal, and post-pubertal stages (*n* = 5). N = 4 studies used the age group of 18 to 32 for assessing the reliability of digital cephalometry in age assessment in young adults, while Lindner et al. included populations of all age groups ranging from 7 to 76 years. The mean age of the subjects in all the studies, however, ranged from 13 to 27 years.

The number of females was significantly higher as compared to males in the majority of the studies. This could be because females show a greater concern for orofacial esthetics and, therefore, opt more commonly for orthodontic treatment as compared to males. Particularly, young females are the category of patients who most often apply for orthodontic treatment, probably because of their higher aesthetic demands, despite their objective needs being no greater. Manual tracing of cephalograms has always been performed using lead acetate paper (0.03″) and a lead pencil, as noted across the various studies included in the present systematic review. Additional equipment utilized in manual cephalometry includes rulers, protractors, tapes, and other stationary.

In the present day, various softwares are available to perform digital cephalometric analysis, which were utilized across the different studies included in the present systematic review. These included FACAD (*n* = 5), Dolphin (*n* = 4), Webceph (*n* = 3), AutoCeph (*n* = 2), OneCeph (*n* = 2), NemoCeph, and SmileCeph. While the majority of the studies compared digital cephalometry with manual tracing as a control, some authors also compared two different softwares; for instance, one study compared Dolphin to AutoCeph, and two studies compared WebCeph to FACAD [29,36,40]. With the advent of AI-based software systems, recent investigators have tested and compared their utility against conventional computer software [36,40,41]. These researchers found that while AI-based software offers various advantages such as comfort, practicality, and speed, further research is crucial before declaring them enough to replace the adequately tested computer software.

When considering the conclusive findings reported by the authors of the studies included in the present systematic review, the majority found digital cephalometry more preferable and reliable as compared to manual tracing, offering additional advantages such as being reliable, rapid, accurate, user-friendly, time-saving, portable, and cloud-based archiving. One author concluded that “the results obtained for manual and digital were almost similar, but the digital landmark plotting has an added advantage in archiving, retrieval, and transmission and can be enhanced during the plotting of lateral cephalograms [30]”. Another study found tablet-based digital cephalometry to be equally reliable as computer-based digital cephalometry and manual tracing [23]. Other *n* = 4 studies also found digital cephalometry to have the same accuracy and reliability as the manual method, suggesting that it can readily replace the conventional cephalometry technique. Only one study performed in Thailand found that digital cephalometry was not as reliable as manual analysis and that it should only be used to support a diagnosis rather than as a sole diagnostic tool [31].

### 3.2. Meta-Analysis

A meta-analysis was performed to synthesize the findings of the studies comparing digital cephalometry to manual cephalometry. The data synthesis utilized both descriptive and quantitative synthesis approaches to provide a comprehensive overview of the studies included in this analysis.

#### 3.2.1. Effect Measures

Effect measures are essential statistical constructs used to compare outcome data between two intervention groups. Examples include odds ratios and mean differences, which assess the odds of an event and the differences in mean values between groups, respectively. For this study, mean and standard deviation values were used as effect measures.

#### 3.2.2. Study Inclusion

The meta-analysis incorporated data from a total of 14 studies. In a study conducted by Mahato et al. in 2016 [29], two different software methods, AutoCEPH and Dolphin, were utilized for digital tracing. For our quantitative assessment, data from both methods were considered, and the study was subdivided into two distinct comparisons: Mahato 2016 (A) for AutoCEPH vs. manual tracing and Mahato 2016 (B) for Dolphin vs. manual tracing.

#### 3.2.3. Maxilla

1. SNA (Sella-Nasion-A Point): Our meta-analysis included data from twelve studies for the assessment of SNA. The pooled SNA estimate was 0.54 (95% CI: −0.28 to 1.35), suggesting that SNA values were greater with digital tracing compared to manual tracing. However, the overall results were not statistically significant (*p* > 0.05), and there was substantial heterogeneity (65%), necessitating the use of a random effects model for analysis.
2. Co-A (Cephalometric A Point): Our analysis incorporated data from five studies for Co-A measurements, resulting in a pooled value of 0.78 mm (95% CI: −1.37 to 2.94). This indicates that Co-A measurements were greater with digital tracing compared to manual tracing. Similarly, the overall results were not statistically significant (*p* > 0.05), with a high level of heterogeneity (89%), leading to the application of a random effects model.
3. Nperp-A (Nasion Perpendicular A): Our analysis included data from two studies for Nperp-A measurements, resulting in a pooled value of −2.30 mm (95% CI: −4.11 to −0.50), indicating that Nperp-A measurements were smaller with digital tracing compared to manual tracing. Notably, the overall results were not statistically significant (*p* > 0.05), and heterogeneity was minimal (0%).

**Figure 4:**
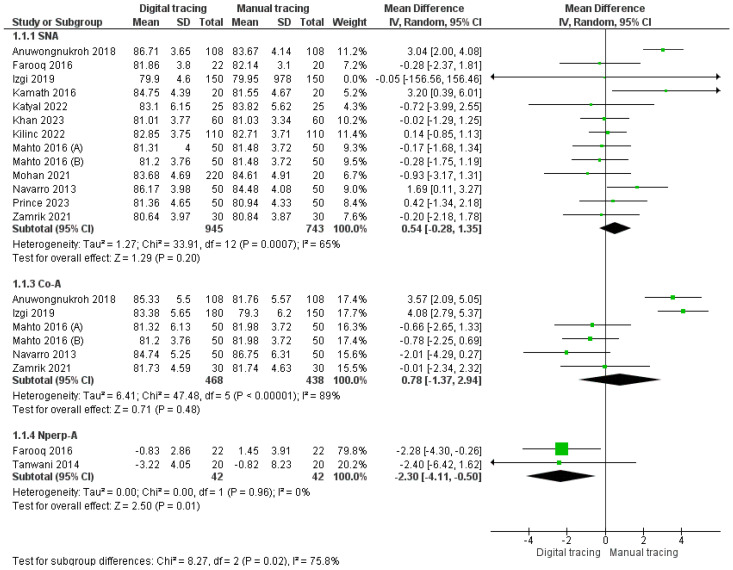
Forest plot for maxilla landmarks [22,25,26,27,29,31,33,34,35,36,37,39,41].

The forest plot for maxillary landmarks is depicted in Figure 4.

#### 3.2.4. Mandible

1. SNB (Sella-Nasion-B Point): Eleven studies were incorporated into the assessment of SNB. The pooled SNB estimate was 0.26 (95% CI: −0.43 to 0.95), suggesting that SNB values were greater with digital tracing compared to manual tracing. Nevertheless, the overall results were not statistically significant (*p* > 0.05), and there was a moderate level of heterogeneity (39%), leading to the use of a random effects model for analysis.
2. Co-Gn (Cephalometric Gnathion): Our analysis included data from five studies for the evaluation of Co-Gn measurements. The pooled Co-Gn estimate was −0.39 (95% CI: −1.69 to 0.90), indicating that Co-Gn measurements were smaller with digital tracing compared to manual tracing. The overall results were not statistically significant (*p* > 0.05), with a low level of heterogeneity (8%).
3. Pog-NB: Two studies were included in the assessment of Pog-NB. The pooled value obtained was 2.91 [−3.58, 9.40], which was greater with digital tracing as compared to manual tracing. Overall results were not statistically significant (*p* > 0.05) with 100% heterogeneity. As a result, the random effects model was used for analysis.
4. FMPA: Eight studies were included in the assessment of FMPA. The pooled value obtained was 0.62 [−0.54, 1.78], which was greater with digital tracing as compared to manual tracing. Overall results were not statistically significant (*p* > 0.05) with 50% heterogeneity. As a result, the random effects model was used for analysis.
5. MIA: Two studies were included in the assessment of FMIA. The pooled mean difference value obtained was −0.28 [−2.92, 2.37], which was less with digital tracing as compared to manual tracing. Overall results were not statistically significant (*p* > 0.05) with 0% heterogeneity.
6. Nperp-Pog: Two studies were included in the assessment of Nperp-Pog. The pooled value obtained was −4.41 [−9.07, 0.26], indicating that Nperp-Pog was less with digital tracing as compared to manual tracing. Overall results were not statistically significant (*p* > 0.05), with 24% heterogeneity.

**Figure 5:**
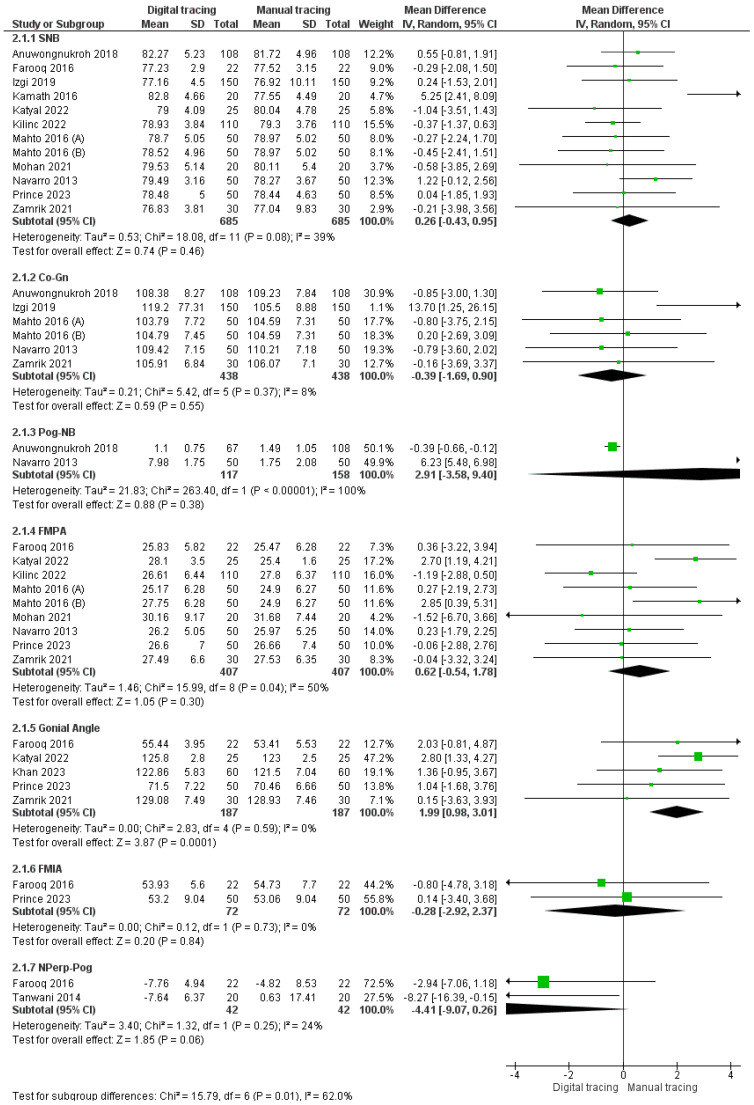
Forest plot for mandible landmarks [22,25,26,27,29,31,33,34,35,36,37,39,41].

The forest plot for mandibular landmarks is depicted in Figure 5.

#### 3.2.5. Intermaxillary Relationships

1. ANB: Ten studies were included in the assessment of the ANB angle. The pooled value obtained was −2.29 [−4.66, 0.06], indicating that ANB was lower with digital tracing as compared to manual tracing. Overall results were not statistically significant (*p* > 0.05), with 97% heterogeneity. As a result, a random effects model was used for analysis.
2. Wits appraisal: Four studies were included in the assessment of Wits appraisal. The pooled value obtained was −0.28 [−1.08, 0.51]. This implies that the value of the Wits appraisal obtained with digital tracing was less than manual tracing. Overall results were not statistically significant (*p* > 0.05) with 0% heterogeneity.
3. ANS-Me: Five studies were included in the assessment of ANS-Me landmark. The pooled value obtained was 0.85 [−0.28, 2.28]. This implies that the value of ANS-Me obtained with digital tracing was greater than that obtained with manual tracing. Overall results were not statistically significant (*p* > 0.05) with 55% heterogeneity. A random effects model was used for analysis.
4. Jarabak ratio: Two studies were included in the assessment of the Jarabak ratio. The pooled value obtained was −0.11 [−1.39, 1.18]. This implies that the value of the Jarabak ratio obtained with digital tracing was less than that obtained with manual tracing. Overall results were not statistically significant (*p* > 0.05) with 0% heterogeneity.

**Figure 6:**
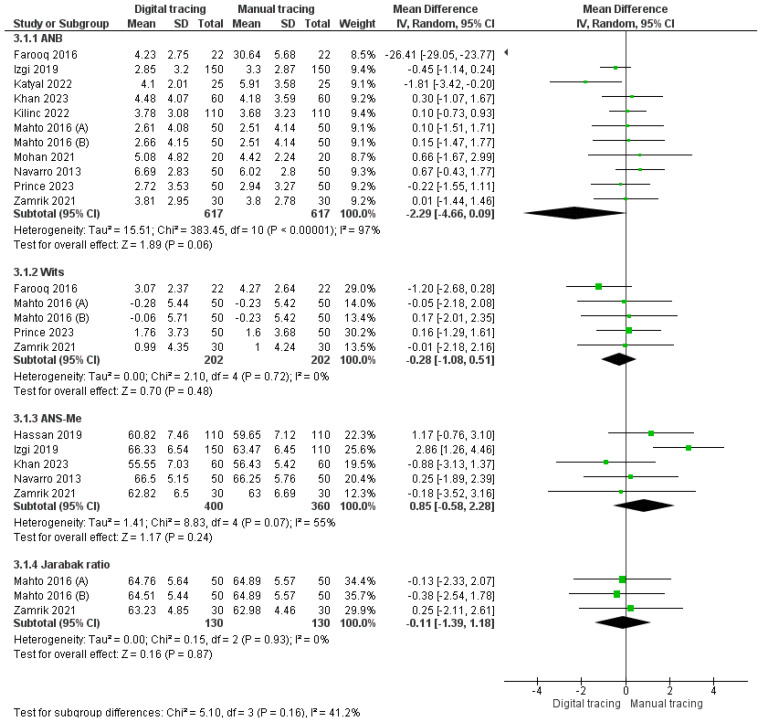
Forest plot for intermaxillary relationships [22,25,26,27,29,31,33,34,35,36,37,39,41].

The forest plot for intermaxillary relationships is depicted in Figure 6.

#### 3.2.6. Dentoalveolar

1. U1-A point: Two studies were included in the assessment of the U1-A point landmark. The pooled value obtained was −0.24 [−0.73, 0.24], indicating that the value of this landmark obtained with digital tracing was less as compared to manual tracing. Overall results were not statistically significant (*p* > 0.05), with 32% heterogeneity. As a result, a random effects model was used for analysis.
2. LI-A Pog: Four studies were included in the assessment of the LI-A Pog landmark. The pooled value obtained was −0.15 [−0.38–0.07], indicating that the value of this landmark obtained with digital tracing was less as compared to manual tracing. Overall results were not statistically significant (*p* > 0.05), with 21% heterogeneity.
3. IMPA: Five studies were included in the assessment of the IMPA angle. The pooled value obtained was −0.67 [−2.69, 1.34]. The value of this landmark obtained with digital tracing was less as compared to manual tracing. Overall results were not statistically significant (*p* > 0.05) with 85% heterogeneity. As a result, a random effects model was used for analysis.
4. UI-NA angle: Eight studies were included in the assessment of the UI-NA angle. The pooled value obtained was −0.17 [−0.51–0.17] degrees, indicating that the value of this landmark obtained with digital tracing was less as compared to manual tracing. Overall results were not statistically significant (*p* > 0.05), with 21% heterogeneity.
5. UI-NA (mm): Ten studies were included in the assessment of UI-NA distance. The pooled value obtained was −0.09 [−0.52, 0.34] mm indicating that the value of this landmark obtained with digital tracing was less as compared to manual tracing. Overall results were not statistically significant (*p* > 0.05) with 92% heterogeneity. As a result, a random effects model was used for analysis.
6. LI-NB angle: Nine studies were included in the assessment of the LI-NB angle. The pooled value obtained was −0.09 [−0.27, 0.08] degrees, indicating that the value of this landmark obtained with digital tracing was less as compared to manual tracing. Overall results were not statistically significant (*p* > 0.05), with 43% heterogeneity. As a result, a random effects model was used for analysis.
7. LI-NB (mm): Ten studies were included in the assessment of LI-NB distance. The pooled value obtained was 0.10 [−0.12, 0.31] mm indicating that the value of this landmark obtained with digital tracing was greater as compared to manual tracing. Overall results were not statistically significant (*p* > 0.05) with 70% heterogeneity. As a result, a random effects model was used for analysis.
8. Go Gn to SN: Four studies were included in the assessment of the Go Gn to SN landmark. The pooled value obtained was 0.11 [−0.04, 0.27], indicating that the value of this landmark obtained with digital tracing was greater as compared to manual tracing. Overall results were not statistically significant (*p* > 0.05) with 0% heterogeneity.
9. Nasolabial angle: Six studies were included in the assessment of the Nasolabial angle. The pooled value obtained was 0.24 [−0.05, 0.53] degrees, indicating that the value of this landmark obtained with digital tracing was greater as compared to manual tracing. Overall results were not statistically significant (*p* > 0.05), with 59% heterogeneity. As a result, a random effects model was used for analysis.
10. Interincisal angle: Three studies were included in the assessment of the interincisal angle. The pooled value obtained was −0.03 [−0.27, 0.21] degrees, indicating that the value of this landmark obtained with digital tracing was less as compared to manual tracing. Overall results were not statistically significant (*p* > 0.05) with 0% heterogeneity.
11. LAFH: Two studies were included in the assessment of the LAFH landmark. The pooled value obtained was −0.51 [−1.36, 0.35], indicating that the value of this landmark obtained with digital tracing was less as compared to manual tracing. Overall results were not statistically significant (*p* > 0.05), with 73% heterogeneity. As a result, a random effects model was used for analysis.

## 4. Discussion

The comprehensive review of 20 studies spanning the past decade provides valuable insights into the comparative analysis of digital and manual cephalometry. Digital cephalometry began to emerge in the late 20th century. While there is ambiguity regarding the exact time of its introduction, it gained significant traction in the 1980s and 1990s. It is crucial to acknowledge the temporal dimension in the interpretation of findings, as technological improvements and methodological refinements may have occurred over this time span. The recently introduced software has greater accuracy and reliability, and thus, the present systematic review particularly selected the studies conducted in the past decade so that the findings would be more relevant to the present-day scenario. This was carried out to ensure that the reviewed evidence was of good quality with updated standards of research methodology and clinical practice.

The geographical distribution of these studies reveals a notable concentration of research in certain regions. Predominantly, the majority of studies emanated from India, comprising nearly half of the total sample. This concentration may be attributed to various factors, including the prevalence of cephalometric research initiatives, local expertise, and regional healthcare priorities. The diversity in the geographical origin of studies, encompassing countries such as Turkey, Pakistan, Brazil, Italy, Taiwan, Thailand, and Mexico, introduces a cross-cultural dimension to the analysis. Regional variations in diagnostic practices, patient demographics, and available resources may contribute to nuanced findings and should be considered in the broader context of cephalometric research.

In assessing the implications of these findings, it is essential to recognize that the geographical distribution may influence the generalizability of the results. Cephalometric analyses are inherently sensitive to population-specific characteristics, and the prevalence of certain anatomical variations or craniofacial features may differ across diverse populations. Consequently, the applicability of conclusions drawn from studies in one region to a broader demographic should be approached with caution. Future research endeavors should aim to foster a more globally representative body of literature to enhance the external validity of cephalometric findings.

The observed variation in sample sizes across the reviewed studies ranged from 20 to 150 subjects, with a notable exception being one study with a substantial sample size of 400 subjects in the Taiwanese population [28]. The choice of sample size is a critical aspect of study design and can significantly influence the statistical power and generalizability of results [42]. The conventional wisdom in research design emphasizes the importance of adequately powered studies to detect meaningful effects and enhance the external validity of findings.

The relatively smaller sample sizes in the majority of Indian studies, ranging from 20 to 50 subjects, underscore a potential limitation in the representativeness of these findings, particularly given the vast and ethnically diverse population of India. Notably, the study by Khattri et al. [40] stands out with a larger sample size of 100 subjects. The decision to adopt a larger sample size in this instance may reflect an awareness of the need for increased statistical power to draw robust conclusions, acknowledging the demographic intricacies within India.

Given the larger population size and ethnic diversity across the various states of India, it is prudent to advocate for studies with relatively larger sample sizes conducted across different geographical areas. This recommendation is rooted in the understanding that a more expansive and diverse sample allows for a more reliable exploration of cephalometric variations within the Indian population. The call for larger sample sizes is particularly relevant in the context of cephalometry, where subtle anatomical differences may exist across diverse ethnic groups.

The incorporation of diverse age groups in the evaluated studies underscores the multifaceted nature of cephalometric analysis, particularly in the context of age estimation methods aimed at identifying the pubertal growth spurt [43]. The inclusion of patients spanning pre-pubertal, pubertal, and post-pubertal stages in a substantial number of studies (*n* = 5) aligns with the inherent focus on capturing the dynamic changes associated with facial and craniofacial development during adolescence [44].

A subset of studies (*n* = 4) specifically targeted young adults, utilizing the age group of 18 to 32 years for assessing the reliability of digital cephalometry in age estimation. This focused age range is strategically chosen to encompass the critical period of post-pubertal growth and maturation, allowing for a detailed examination of cephalometric parameters during this transitional phase. The decision to concentrate on young adults recognizes the clinical relevance of age estimation in orthodontic and maxillofacial contexts, where the assessment of skeletal maturity plays a pivotal role in treatment planning.

The study by Lindner et al. [28] stands out for its inclusivity, encompassing populations of all age groups ranging from 7 to 76 years. This broad age spectrum is noteworthy as it extends the applicability of digital cephalometry beyond the conventional focus on adolescent and young adult populations. The inclusion of older individuals in cephalometric studies addresses the potential impact of aging on craniofacial structures and provides insights into the utility of digital cephalometry across the entire lifespan. This comprehensive age representation is particularly relevant for clinical scenarios where cephalometric analysis may be applied to individuals of varying ages.

Despite the diversity in the age groups studied, the mean age of subjects across all included studies consistently ranged from 13 to 27 years. This convergence around a relatively narrow age range reflects a common emphasis on the critical period of facial growth and development. The decision to focus on this age range may be driven by the recognition that the pubertal growth spurt, a key aspect of age estimation, is most pronounced during adolescence.

The observed predominance of females over males in the majority of the reviewed studies raises intriguing considerations regarding gender distribution in cephalometric research. The higher representation of females could be attributed to multifaceted factors, with one plausible explanation being the heightened concern among females towards orofacial esthetics [45]. This inclination is consistent with existing literature suggesting that females often exhibit a greater awareness of and emphasis on facial appearance and dental aesthetics.

The phenomenon of a greater female representation in orthodontic studies aligns with broader trends in healthcare-seeking behavior [46]. It is well documented that females tend to be more proactive in seeking orthodontic treatment, possibly due to their heightened aesthetic awareness and societal expectations [47,48]. The perception of orthodontic treatment as a means to enhance facial esthetics may contribute to the increased prevalence of females in these studies. The observed gender disparity may reflect not only the prevalence of orthodontic issues among females but also their proactive approach to addressing these concerns.

It is essential to acknowledge that the gender distribution in cephalometric studies may introduce a potential bias in the generalizability of findings. Cephalometric analyses are inherently sensitive to gender-specific anatomical variations, and an overrepresentation of females may skew results towards characteristics more prevalent in that demographic. Consequently, the external validity of cephalometric conclusions, especially in the context of treatment planning, should be interpreted with consideration for the gender bias inherent in the available literature.

The consistent use of lead acetate paper (0.03″) and lead pencil in manual cephalometry, as documented across the various studies included in this systematic review, highlights the traditional methods and materials employed in this technique. The utilization of lead acetate paper with a specific thickness of 0.03″ speaks to the standardization and precision required in manual tracing to ensure accurate cephalometric measurements [10,49]. The tactile feedback and ease of marking provided by lead acetate paper contribute to the reliability of manual cephalometric tracings.

In addition to lead acetate paper and lead pencils, the mention of supplementary equipment such as rulers, protractors, tapes, and other stationary items underscores the meticulous nature of manual cephalometry [10,14]. These tools are essential for the precise measurement of angles, distances, and anatomical landmarks on cephalograms. Rulers and protractors aid in maintaining consistency in measurements, while tapes may be employed for linear assessments. The comprehensive set of stationary tools reflects the thorough approach required for manual cephalometric analysis, where even subtle deviations in measurements can have clinical implications.

Among the software applications mentioned, FACAD emerges as one of the most frequently employed tools, with five studies incorporating its use. Dolphin, Webceph, AutoCeph, and OneCeph also contribute to the digital cephalometric landscape, each being utilized in multiple studies [22,23,24,25,26,27,28,29,30,31,32,33,34,35,36,37,38,39,40,41]. The choice of software may be influenced by factors such as user familiarity, interface capabilities, and specific features tailored to the requirements of cephalometric analysis [50].

Noteworthy is the comparative aspect of certain studies, where researchers have not only contrasted digital cephalometry with manual tracing but have also directly compared different software platforms [29,36,40]. These intra-digital software comparisons offer valuable insights into the nuanced differences between platforms and contribute to the ongoing refinement of digital cephalometric methodologies. The recent exploration of AI-based software systems in cephalometric analysis marks a notable advancement in the field [36,40,41]. These studies acknowledge the advantages offered by AI-based tools, such as increased comfort, practicality, and speed. The potential of AI to automate landmark identification and streamline the analysis process represents a paradigm shift towards more efficient and possibly more accurate cephalometric assessments.

However, the cautious stance adopted by researchers, emphasizing the need for further research before considering AI-based software as a replacement for established computer software, underscores the importance of rigorous validation and scrutiny in the integration of new technologies. The dynamic nature of cephalometric analysis, coupled with the intricate nature of craniofacial anatomy, necessitates a thorough evaluation of the capabilities and limitations of AI-based systems to ensure their reliability and clinical applicability.

The analysis of intermaxillary relationships provided insights into parameters that assess the relative positions of the maxilla and mandible. The ANB angle, a significant indicator of anteroposterior jaw relationships, displayed lower values with digital tracing. The pooled estimate of −2.29 was statistically significant (*p* < 0.05), with high heterogeneity (97%), indicating that digital tracing may provide more precise results for the ANB angle. Wits appraisal, a parameter that helps in assessing the relationship between the maxilla and mandible in three dimensions, demonstrated lower values with digital tracing, but the overall results were not statistically significant (*p* > 0.05). The lack of heterogeneity (0%) within these studies suggests consistent outcomes for Wits appraisal measurements.

ANS-Me, which evaluates the vertical relationship of the maxilla and mandible, presented a trend with digital tracing yielding higher values, although the overall results were not statistically significant. The moderate heterogeneity (55%) within this group of studies emphasizes the importance of considering variations in the software and measurement techniques used for digital cephalometry. Conversely, the Jarabak ratio demonstrated smaller values with digital tracing, and the results were not statistically significant. Furthermore, the heterogeneity was low (0%), implying consistent outcomes for this parameter between digital and manual tracing [51].

The meta-analysis provides an extensive evaluation of digital cephalometry compared to manual tracing in orthodontics. The findings suggest that digital tracing shows promise in providing reliable measurements for specific cephalometric parameters. However, substantial heterogeneity among studies highlights the need for standardization in software, techniques, and measurements. Further research is necessary to determine the clinical significance of these differences and to better guide the choice of tracing methods in orthodontic practice. The potential benefits of digital cephalometry in terms of time-saving and user-friendliness should also be taken into account, as they may impact clinical workflow and patient care [52].

The collective findings of the studies included in the systematic review present a compelling argument in favor of digital cephalometry, with the majority of authors reporting it as more preferable and reliable compared to manual tracing. These conclusive statements are supported by a spectrum of advantages attributed to digital cephalometry, ranging from reliability and accuracy to practical benefits such as speed, user-friendliness, portability, and cloud-based archiving. The recognition of digital cephalometry’s potential for enhancement during the plotting of lateral cephalograms suggests a transformative role in streamlining workflows and improving overall diagnostic efficiency.

One study contributed to the consensus by finding tablet-based digital cephalometry to be equally reliable as computer-based digital cephalometry and manual tracing [23]. This result underscores the versatility of digital cephalometry, as it extends beyond computer-based platforms to accommodate emerging technologies like tablets. The equivalence in reliability further supports the notion that digital cephalometry can be seamlessly integrated into established diagnostic protocols.

Four additional studies, aligning with the overarching trend, report that digital cephalometry exhibits equal accuracy and reliability as the manual method. This collective sentiment echoes the idea that digital cephalometry has reached a level of maturity and precision comparable to traditional manual tracing, suggesting its readiness for widespread adoption in clinical practice. The implication is that digital cephalometry has the potential to supplant conventional methods, offering a more efficient and technologically advanced alternative.

It is noteworthy that the study in Thailand presents a dissenting perspective, noting that digital cephalometry was not as reliable as manual analysis [31]. The cautious conclusion, suggesting that digital cephalometry should be used to support a diagnosis rather than as a sole diagnostic tool, highlights the importance of considering regional and contextual variations in the adoption of new technologies. This dissenting view also underscores the need for ongoing research to address potential challenges and refine digital cephalometric methodologies.

The limitations of our analysis include the heterogeneity in software, study design, and sample characteristics, which may have influenced the results. Future studies should aim to address these limitations and provide more robust evidence on the advantages and disadvantages of digital cephalometry in orthodontics. Nevertheless, our findings suggest that digital cephalometry has the potential to enhance clinical practice by offering consistent and user-friendly alternatives to traditional manual tracing techniques.

## 5. Conclusions

The present meta-analysis compared digital cephalometry to manual cephalometry in orthodontics, revealing trends suggesting that digital tracing may offer reliable measurements for specific cephalometric parameters. Based on the comprehensive analysis of twenty studies conducted between 2013 and 2023 comparing manual and digital cephalometric tracing methods, our systematic review reveals varied outcomes across different cephalometric landmarks. While digital tracing generally demonstrated increased measurements for maxillary landmarks such as SNA and Co-A, the differences were not statistically significant, indicating comparable accuracy to manual tracing. Conversely, mandibular landmarks, including SNB and Co-Gn, exhibited greater measurements with digital tracing, albeit without statistical significance. Notably, some landmarks like Nperp-A and Pog-NB displayed smaller measurements with digital tracing, though again lacking statistical significance. Moreover, intermaxillary relationships, as assessed by ANB and Wits appraisal, showed trends towards smaller measurements with digital tracing, while ANS-Me displayed larger measurements. Dentoalveolar landmarks exhibited mixed results, with some showing smaller measurements with digital tracing (e.g., U1-A point, IMPA) and others displaying greater measurements (e.g., LI-NB distance, Go Gn to SN). Importantly, none of the observed differences reached statistical significance, suggesting that digital cephalometry, while offering potential advantages such as enhanced efficiency and reduced operator bias, does not significantly alter measurement outcomes compared to manual methods. Thus, both approaches remain valid options, and the choice between them may depend on factors such as resource availability, expertise, and workflow preferences.

However, substantial heterogeneity among studies highlights the need for standardization in software, techniques, and measurements. Further research is required to determine the clinical significance of these differences and to better guide the choice of tracing methods in orthodontic practice. Additionally, orthodontists must consider the potential benefits of digital cephalometry, including time-saving and user-friendliness, and how they may impact clinical workflow and patient care. Despite the need for further exploration and standardization, the potential of digital cephalometry to enhance clinical practice is a promising development in the field of orthodontics.

## Figures and Tables

**Table 1:** Characteristics and findings of the studies included in the present systematic review.

Sr. No.	Author and Year of Study	Country	Sample Size	Age Range (in Years)	Gender (M/F)	Software Used for Digital Cephalometry	Comparator Technique Short Description	Conclusive Findings
1.	Navarro 2013 [22]	Brazil	50	NP	NP	Dolphin Imaging 11 program i-Cat tomography	Conventional manual tracing	Digital cephalometry: Reliable, similar to manual tracing
2.	Goracci 2014 [23]	Italy	20	NP	NP	NemoCeph NX 2009 SmileCeph	Images rescaled to 1:1 using Adobe Photoshop CS and printed on semi-gloss paper on a clear acetate sheet placed over printed images by lead pencil	Tablet-assisted cephalometry: Comparable to manual and PC-aided methods; preferred when user-friendliness and portability are prioritized
3.	Iacob 2014 [24]	Romania	60	8 to 23	22/38	Orthalis cephalometric software	Manual tracing using a 0.5 mm pen on a 0.003-inch acetate paper on a light box, in a dark room	Digital cephalometry: Accuracy akin to manual technique
4.	Tanwani 2014 [25]	India	20	NP	NP	Dolphin imaging v 11.7.5.55	A sheet of lead acetate tracing paper measuring 8 × 10-in and 0.003-in thickness on a view box with the tracing paper positioned over the radiograph with masking tape	Manual vs. digital tracings: Statistically significant differences in Burstone and McNamara’s analyses
5.	Farooq 2016 [26]	India	44	17 to 30	NP	FACAD 3.6 software	Images resized to 1:1 scale using Adobe Photoshop CS and printed on semi-gloss paper. Traced using a lead pencil on a clear acetate sheet placed over printed images	Most of the commonly used measurements made by digital cephalometry were accurate
6.	Kamath 2016 [27]	India	20	NP	NP	FACAD software Ilexis AB, Linköping,	Traced on a view box with acetate tracing paper securely positioned over the radiograph with masking tape.	Manual and digital cephalometry showed statistically significant differences in the measurements obtained on performing Steiner’s analysis
7.	Lindner 2016 [28]	Taiwan	400	7 to 76 mean: 27	165/235	FALA system	Manual tracing	Digital cephalometry: Enhances clinical workflow efficiency by rapidly and accurately analyzing cephalometric landmarks
8.	Mahto 2016 [29]	India	50	NP	NP	AutoCEPH© version 1.0 Dolphin^®^ imaging software 11.7	Using a millimeter ruler and protractor	Digital cephalometry: Agreement with manual tracing, suitable for routine analysis
9.	Kasinathan 2017 [30]	India	50	NP	NP	Dolphin Imaging v 11.8	0.5 mm lead pencil on a 0.003 thickness acetate sheet in a dark room over an X-ray view box	Digital cephalometry: Similar results to manual, with advantages in archiving and transmission
10.	Anuwongnukroh 2018 [31]	Thailand	108	NP	NP	Carestream Dental V6.14	Manual tracing by overlaying acetate papers on lateral cephalograms	Digital cephalometry: Not as reliable as manual, best used to support diagnosis
11.	Hassan 2019 [32]	Pakistan	110	18 to 38 mean: 23.43	44/66	TrophyDicom software	Lead pencil in a dark room on an illuminator.	Digital cephalometry: User-friendly, time-saving alternative to manual tracing
12.	Izgi 2019 [33]	Turkey	150	12 to 34	75/75	OnyxCeph V3.1.54	0.3 mm 2H lead pencil, a ruler, and a protractor on an A4 paper placed over the printed image	Digital cephalometry: Preferred over manual method
13.	Mohan 2021 [34]	India	20	18 to 32 mean: 22.4	20/20	OneCeph	0.3 mm lead pencil on a sheet of fine grade 36 μm matte acetate tracing paper taped over the X-ray printout	Digital cephalometry: Reliable, fast, and practical for clinical use OneCeph is a simple, reliable, accurate alternative to manual tracing that saves clinical time and armamentarium.
14.	Zamrik 2021 [35]	Turkey	30	NP	NP	OneCeph	Manual tracing using a 0.3 mm hard black (HB) lead pencil	Digital vs. manual cephalometry: Clinically insignificant differences. Both tracing methods reliable for daily clinical practice.
15.	Katyal 2022 [36]	India	25	mean 18	14/11	WebCeph FACAD	Digital images were imported to Adobe Photoshop 7.0 and rescaled to 1:1, then printed Manually traced using a 0.35 mm lead pencil	Digital cephalometry: Reliable, with advantages of online AI-based software (WebCeph) including cloud-based storage, online archiving, quick analysis, no need for specific installation or software, and compatibility with any operating system.
16.	Klinic 2022 [37]	Turkey	110	10 to 24 mean: 15.83	44/66	SATM CephNinja V4.20 WebCeph	Manual tracing using a 0.3 mm hard black lead pencil	Digital vs. manual cephalometry: Statistically and clinically significant differences Digital cephalometry on smartphones: Clearer image perception, improved comfort AI-based cephalometry: Promises enhanced comfort, practicality, speed
17.	Salgado 2022 [38]	Mexico	42	7 to 19 mean: 13	18/24	Cephalopoint	4H pencil, adhesive tape, protractor, ruler, erasers, tracing paper, and negatoscope,	Digital cephalometry: One-third time of manual tracing, efficient analysis
18.	Khan 2023 [39]	Pakistan	120	12 to 24 mean: 17.37	56/64	View Box V4.0	0.5 mm lead pencil and protractors on 0.003-inch matte acetate paper under a standard view box	No significant difference: Manual vs. digital cephalometry for selected angular and linear measurements
19.	Khattri 2023 [40]	India	100	NP	NP	WebCeph V15.0 FACAD	Manual tracing	AI-based tracing: Not yet ready to replace semi-automated computer-aided methods
20.	Prince 2023 [41]	India	50	NP	NP	AutoCEPH©	The cephalograms were printed on 8 × 10-in size radiographic film using a compatible X-ray printer.	WebCeph™ AI software: High agreement with validated methods—AutoCEPH© and manual tracing.

NP = Not provided.

**Table 2:** Methodological quality appraisal of included studies.

Study ID	Objective Clearly Stated?	Study Population Clearly Defined?	Participation Rate at Least 50%?	Subjects Comparable?	Justification of Sample Size?	Reliability of Outcome Measures?	Assessors Blinding?	Adjustment for Confounders?	Quality of Studies
Navarro 2013 [22]	Yes	Yes	Yes	Yes	Yes	Yes	No	No	Good
Goracci 2014 [23]	Yes	Yes	Yes	Yes	No	Yes	No	No	Good
Iacob 2014 [24]	Yes	Yes	Yes	Yes	No	Yes	No	No	Good
Tanwani 2014 [25]	Yes	Yes	Unclear	Yes	No	Yes	Unclear	No	Fair
Farooq 2016 [26]	Yes	Yes	Yes	Yes	No	Yes	No	No	Good
Kamath 2016 [27]	Yes	Yes	Unclear	Yes	No	Yes	No	No	Fair
Lindner 2016 [28]	Yes	Yes	Yes	Yes	No	Yes	No	No	Good
Mahto 2016 [29]	Yes	Yes	Yes	Yes	No	Yes	No	No	Good
Kasinathan 2017 [30]	Yes	Yes	Unclear	Yes	No	Yes	No	No	Fair
Anuwongnukro 2018 [31]	Yes	Yes	Yes	Yes	No	Yes	No	Yes	Good
Hassan 2019 [32]	Yes	Yes	Yes	Yes	No	Yes	No	No	Good
Izgi 2019 [33]	Yes	Yes	Yes	Yes	No	Yes	No	No	Good
Mohan 2021 [34]	Yes	Yes	Yes	Yes	No	Yes	No	No	Good
Zamrik 2021 [35]	Yes	Yes	Yes	Yes	No	Yes	No	No	Good
Katyal 2022 [36]	Yes	Yes	Yes	Yes	No	Yes	No	No	Good
Klinic 2022 [37]	Yes	Yes	Yes	Yes	No	Yes	No	No	Good
Salgado 2022 [38]	No	Unclear	Yes	Unclear	No	Yes	No	No	Poor
Khan 2023 [39]	Yes	Yes	Yes	Yes	Yes	Yes	Unclear	No	Good
Khattri 2023 [40]	Yes	Yes	Yes	Yes	No	Yes	Unclear	No	Good
Prince 2023 [41]	Yes	Yes	Yes	Yes	No	Yes	No	No	Good

## Data Availability

The data that supports the findings of the present systematic review are available within the article in the form of tables and forest plots.
